# Malignancy within a Tail Gut Cyst: A Case of Retrorectal Carcinoid Tumour

**DOI:** 10.1155/2014/454502

**Published:** 2014-11-13

**Authors:** A. A. Abukar, B. J. Parcell, C. B. Lim, P. V. Patil, A. Ramsanahie, F. Carey, R. J. C. Steele, M. A. Thaha

**Affiliations:** ^1^Academic Surgical Unit, Blizard Institute, National Centre for Bowel Research & Surgical Innovations, Queen Mary University of London and The Royal London Hospital, Barts Health NHS Trust, London E1 2AT, UK; ^2^Department of Surgery & Molecular Oncology, Ninewells Hospital & Medical School, University of Dundee, Dundee DD1 9SY, UK; ^3^Department of Molecular & Cellular Pathology, Ninewells Hospital & Medical School, University of Dundee, Dundee DD1 9SY, UK

## Abstract

*Purpose.* Tailgut cysts with malignant transformation are rare entities. We discuss the diagnostic strategy and treatment of a malignancy within a tailgut cyst. *Methods.* In this study we report on the case of a 61-year-old man with a malignant neuroendocrine tumour arising within a tailgut cyst and an overview of the literature emphasising the histopathological characteristics and differential diagnosis. *Results.* Our patient presented with lower back pain, rectal pain, and increased urgency of defecation. MRI scan and CT-guided biopsy on histological analysis revealed a diagnosis of carcinoid tumour of the presacral space. The patient subsequently underwent an abdominoperineal excision of the rectum. *Conclusions.* This case highlights the importance of tailgut cysts as a differential diagnosis of presacral masses. It is a rare congenital lesion developing from remnants of the embryonic postanal gut and is predominantly benign in nature. Approximately half of cases remain asymptomatic; therefore, diagnosis is often delayed. Magnetic resonance imaging is the investigation of choice and an awareness of the possibility of malignant potential is critical to avoiding missed diagnosis and subsequent morbidity. Complete surgical excision allows accurate diagnosis, confirmation of oncological clearance, and prevention of mortality.

## 1. Introduction

Tailgut cysts are rare developmental cysts, which occur in the presacral region. They have also been described as retrorectal cysts, cyst of postanal intestine, retrorectal cystic hamartomas, simple cysts, mucus secreting cysts, and myoepithelial hamartoma of the rectum [1–3]. It is rare for carcinoid tumours to be present in the presacral region. If this occurs, it is usually due to direct or metastatic spread from a primary rectal tumour [4–6].

Approximately 23 cases of malignant transformation in tailgut cysts have been reported in the literature, of which eleven have been carcinoid tumours. They are mainly reported in middle-aged women, are usually asymptomatic, and are found during routine physical examination or at childbirth; therefore, diagnosis is often delayed. We describe a case in which a 61-year-old man presented with a three-week history of dull aching pain in his lower back, rectal pain, and increased frequency of defecation. Following abdominoperineal excision of the rectum, the presence of a tailgut cyst was established. Arising within this was a low grade epithelial neoplasm with morphological and immunohistochemical evidence of neuroendocrine differentiation.

## 2. Case Report

A 61-year-old man presented with a history of dull aching pain in his lower spine, rectal pain, and increased urgency of defecation lasting for three weeks. Abdominal examination and digital rectal examination were unremarkable. A barium enema showed extrinsic compression of the lower rectum from a presumed low pelvic mass and two persistent filling defects within the descending colon. Colonoscopy showed a pedunculated polyp at 50 cm. Biopsies of this polyp confirmed it to be a tubulovillous adenoma with mild dysplasia. The rectal mucosa was normal. CT-guided biopsy of the presacral mass initially suggested a prostate cancer. However, on review of the histology and scan, a carcinoid tumour of the presacral space was diagnosed. An MRI of the pelvis carried out showed a loculated mass 6 × 4.3 × 4.8 cm, proximal to the lower rectum. The rectal mucosa was found to be intact whilst the serosal surface was inferiorly breached and in direct contact with the mass. The mass displaced the inferior aspect of levator ani laterally and there was evidence of direct extension into muscle on the left side. Staging CT scan did not show any evidence of distant metastases. The patient underwent abdominoperineal excision of the rectum.

Microscopy of the CT-guided biopsies of the presacral mass showed fibrous tissue extensively infiltrated by an epithelial neoplasm. The tumour was moderately to poorly differentiated and had a focally cribriform growth pattern. Despite being described as moderately to poorly differentiated, the tumour had a low Ki-67 index with no necrosis or other features of poor prognosis. Immunohistochemical staining of the tumour cells strongly expressed prostatic acid phosphatase and showed weak patchy CD56 positivity. Furthermore, it revealed strong and diffuse positivity for neuroendocrine markers: synaptophysin and chromogranin. The tumour cells also coexpressed the cytokeratin MNF116 and showed patchy AE1/3 positivity.

**Figure 1:**
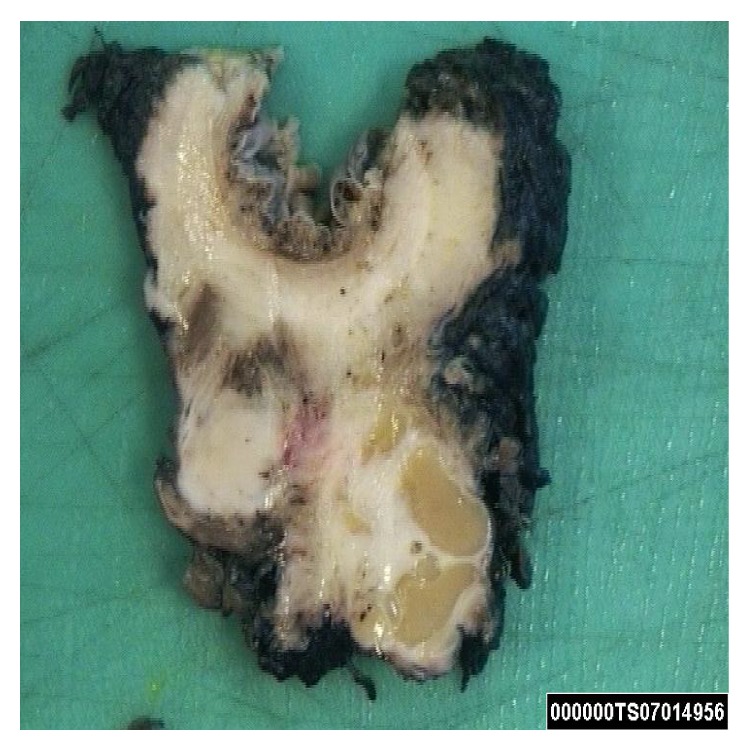
Macroscopic appearance of tumour within the tail gut cyst.

**Figure 2:**
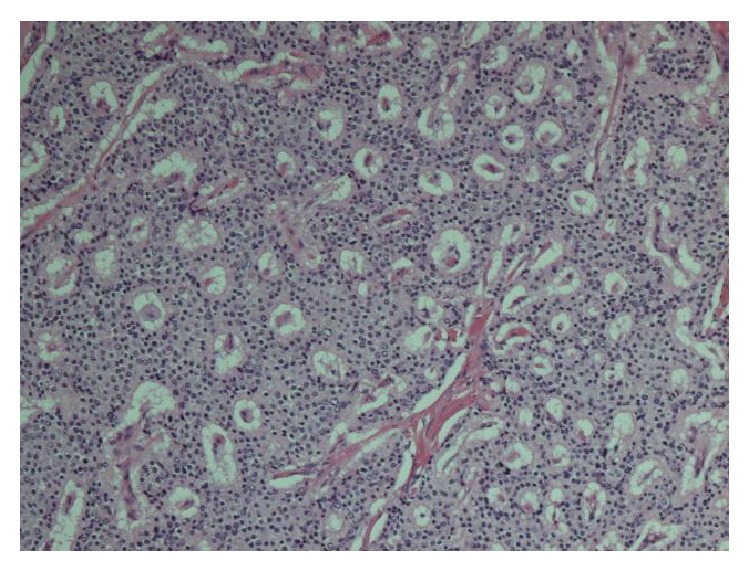
Characteristic microscopic appearance of carcinoid tumour.

**Figure 3:**
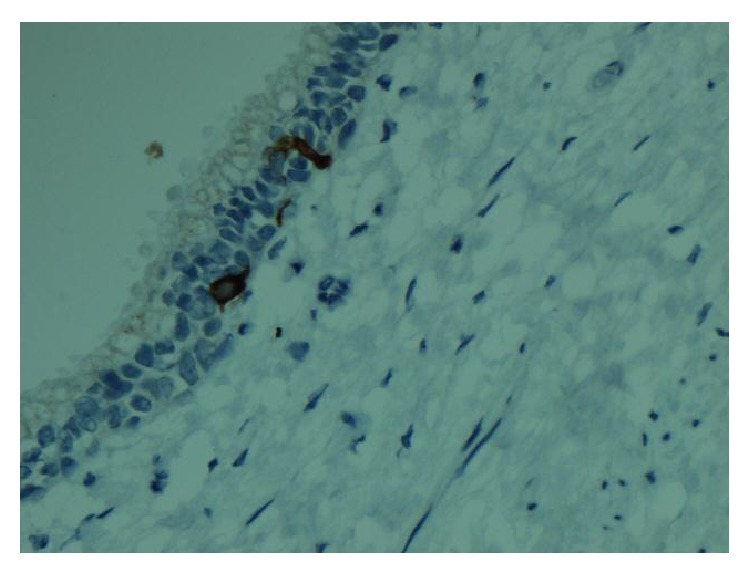
Immunohistochemical staining (columnar lined cells expressing both chromogranin and synaptophysin).

Macroscopically, the surgical resection specimen showed a tumour mass measuring 59 × 42 × 27 mm which was attached posteriorly to the rectum in the midline, close to the distal margin. The tumour surface had a grey-white appearance, and yellow discoloured areas were present (Figure 1). Distally, there were areas of cystic changes within the tumour mass. The tumour involved the muscle of the anal canal but not the mucosa. Microscopic sections of the tumour revealed a homogenous neoplasm that was made up of a uniform population of round cells that had slightly granular nuclei (Figure 2). The cells were arranged in trabeculae and islands with a prominent vascular connective tissue stroma. In some areas, the tumour intermingled with smooth muscle bundles from around the anal canal. The morphological features suggested a neuroendocrine (carcinoid) tumour (Figure 3). This was confirmed by immunohistochemistry which showed that neoplastic cells expressed both chromogranin and synaptophysin. The cysts described inferiorly were lined by columnar mucinous epithelium, which strongly expressed cytokeratins 7 and 19 but was negative for cytokeratin 20. There was a subpopulation of neuroendocrine cells within the cyst lining showing expression of chromogranin and synaptophysin and thus confirming the diagnosis of a carcinoid tumour arising from a retrorectal cystic hamartoma (tailgut cyst). There was no cytological evidence of high grade malignancy; however, the tumour did reach circumferential margin at many points. Proximal lymph nodes from the mesorectum showed no evidence of malignancy. A NM MIBG scan also ruled out distant metastasis.

## 3. Discussion

Tailgut cysts are rare congenital retrorectal lesions. They are thought to develop from the remnant of the tailgut (postanal gut), a primitive gut which is temporarily present at the caudal portion of the embryo [1]. During days 28–35, the embryo possesses a true tail and this is largest on the 35th day of gestation [1, 3]. On the 56th day of gestation, the anus develops above this tail, and by the 8th week the tailgut regresses [1]. It is believed that if this tailgut persists, tailgut cysts may occur [1, 3]. The retrorectal (presacral) space is bounded anteriorly by the rectum and posteriorly by the sacrum [1, 3]. Differential diagnoses of masses in this area include teratomas, dermoid cyst, anal gland cyst, anterior sacral meningocele, and duplication cyst (enterogenous cyst) [1].

Tailgut cysts are found in adults and children but are predominantly reported in middle aged women with a female to male ratio of 3 : 1 [1]. Approximately 50% of patients with tailgut cysts are asymptomatic and are only identified by routine physical examination or at childbirth, and diagnosis is therefore often delayed [1]. Symptoms may be due to mass effects causing abdominal pain, rectal bleeding, tenesmus, and urinary frequency [1, 6, 7]. Tailgut cysts sometimes result in complications such as bleeding, infection, and rarely malignant degeneration [8, 9]. Infection may be misdiagnosed as a pilonidal cyst, recurrent retrorectal abscess, or anorectal fistula [1]. Magnetic resonance imaging is the investigation of choice to image tailgut cysts [10]. Tailgut cysts can result in morbidity and mortality if the mass is not suspected and surgery is not carried out [1]. Tailgut cysts are treated optimally with complete surgical excision, which allows accurate diagnosis and treatment [1].

On macroscopic examination of excised specimens tailgut cysts are typically well circumscribed [1]. These cysts may be multicystic or multiloculated masses with adherent surrounding fibroadipose tissue [1, 8]. The cysts may contain translucent mucoid fluid or clear serous fluid and may be lined by a wide range of epithelia such as stratified squamous type, stratified columnar type, transitional type, cuboidal type, mucinous or ciliated pseudostratified columnar, and gastric type [1, 2, 7, 11]. The majority of cysts contain focal well-formed disorganised bundles of bland smooth muscle cell in their walls [1]. They may develop carcinoid tumours, adenocarcinoma, and germ cell tumours [1]. Carcinoid tumours are thought to develop from neuroendocrine cells present in the glandular epithelium of the tailgut cyst (these cells were prominent in the residual cyst in our case) and can be classified according to their architectural arrangement such as trabecular, glandular, undifferentiated, insular, and mixed [1]. The main differential diagnosis is teratoma and duplication cyst. Our patient had a cyst which did not contain any skin adnexal structures, or mesenchymal tissues, for instance bone and cartilage, excluding the possibility that the cyst was a teratoma. A distinctive feature of duplication cysts is a two-layer smooth muscle bundle wall containing a nerve plexus [11]; this was not identified in our patient. Enteric cysts often show epithelial expression of cytokeratin 20 (absent in our patient).

Diagnosis based on biopsy can be difficult as biopsy specimens commonly contain inflamed fibrous tissue with no epithelia or only one type of epithelium, making it difficult to determine whether the mass is a tailgut cyst or another type of developmental cyst [1]. Malignant foci may be very focal and the sample should be examined carefully. Additionally, malignant cells can spill into the peritoneal cavity during biopsy [6]. In this case, the morphological features of this tumour initially suggested a possible diagnosis of prostate carcinoma. Carcinoid tumours may have a similar histological appearance to prostate cancer and rectal carcinoids may express the marker prostatic acid phosphatase [12, 13]. However, our patient had no clinical evidence of a prostatic mass and PSA was not elevated. In addition, on radiological investigation, the patient's presacral mass also appeared to be separate from the prostate.

This case highlights the importance of tailgut cysts as a differential diagnosis of presacral masses as malignant transformation can occur and result in mortality if surgery is not carried out [1, 6]. Diagnosing tailgut cysts is difficult as their existence is not well recognised, clinical examination may be difficult, and symptoms may mimic other more commonly occurring lesions such as perianal fistulas and abscesses.
